# Application of next-generation sequencing to detect variants of drug-resistant *Mycobacterium tuberculosis*: genotype–phenotype correlation

**DOI:** 10.1186/s12941-018-0300-y

**Published:** 2019-01-03

**Authors:** Dae-Hyun Ko, Eun Jin Lee, Su-Kyung Lee, Han-Sung Kim, So Youn Shin, Jungwon Hyun, Jae-Seok Kim, Wonkeun Song, Hyun Soo Kim

**Affiliations:** 1 Department of Laboratory Medicine, University of Ulsan College of Medicine and Asan Medical Center, Seoul, South Korea; 2 Department of Laboratory Medicine, Hallym University College of Medicine, Chuncheon, South Korea; 3 Korean Institute of Tuberculosis, Cheongju, South Korea; 4 Department of Laboratory Medicine, Hallym University Dongtan Sacred Heart Hospital, Hallym University College of Medicine, 7, Keunjaebong-gil, Hwaseong-Si, Gyeonggi-Do 18450 South Korea

**Keywords:** *Mycobacterium tuberculosis*, Next-generation sequencing, Drug resistance

## Abstract

**Background:**

Drug resistance in *Mycobacterium tuberculosis* (MTB) is a major health issue worldwide. Recently, next-generation sequencing (NGS) technology has begun to be used to detect resistance genes of MTB. We aimed to assess the clinical usefulness of Ion S5 NGS TB research panel for detecting MTB resistance in Korean tuberculosis patients.

**Methods:**

*Mycobacterium tuberculosis* with various drug resistance profiles including susceptible strains (N = 36) were isolated from clinical specimens. Nucleic acids were extracted from inactivated culture medium and underwent amplicon-based NGS to detect resistance variants in eight genes (*gyrA*, *rpoB*, *pncA*, *katG*, *eis*, *rpsL*, *embB*, and *inhA*). Data from previous studies using the same panel were merged to yield pooled sensitivity and specificity values for detecting drug resistance compared to phenotype-based methods.

**Results:**

The sequencing reactions were successful for all samples. A total of 24 variants were considered to be related to resistance, and 6 of them were novel. Agreement between the phenotypic and genotypic results was excellent for isoniazid, rifampicin, and ethambutol, and was poor for streptomycin, amikacin, and kanamycin. The negative predictive values were greater than 97% for all drug classes, while the positive predictive values varied (44% to 100%). There was a possibility that common mutations could not be detected owing to the low coverage.

**Conclusions:**

We successfully applied NGS for genetic analysis of drug resistances in MTB, as well as for susceptible strains. We obtained lists of polymorphisms and possible polymorphisms, which could be used as a guide for future tests applying NGS in mycobacteriology laboratories. When analyzing the results of NGS, coverage analysis of each samples for each gene and benign polymorphisms not related to drug resistance should be considered.

## Background

Tuberculosis (Tb) is an infectious disease caused by *Mycobacterium tuberculosis* (MTB), and is one of the most significant health issues problems worldwide. Approximately 10.4 million incident cases of Tb occurred in 2016; the majority of these were from the South-East Asian Region, followed by the African and Western Pacific Regions. Tb has caused more than 1.6 million deaths worldwide, and is the ninth leading cause of death [1]. Epidemiological control of Tb, especially drug-resistant strains, is one of the most challenging issues globally.

Various methods have been used to detect the susceptibility of MTB to various kinds of drugs, ranging from phenotyping assays to genotyping assays. For phenotyping of drug resistance, proportional methods and absolute concentration methods can be used. Phenotypic methods usually require several weeks to several months to perform, as they require culturing MTB, which is a slow-growing microbe. To overcome this issue, genotypic methods have been used to detect drug resistances in MTB. Line probe assays and the Xpert MTB/RIF assay (Cepheid, Sunnyvale, CA, USA) are representative genotyping methods for detecting drug resistance [2].

There have been several reports using next-generation sequencing (NGS) technology to reveal drug resistance profiles in MTB [3–7]. Previous studies usually used only MTB that was resistant to one or more drugs, without including susceptible strains. In clinical practice, however, it is highly unlikely to encounter such a situation. In other words, clinicians need to apply tests without prior information on the resistance status, and therefore need information on whether variants found in clinical specimens could be related or unrelated to resistance. In that sense, positive predictive value (PPV) and negative predictive value (NPV), as well as sensitivity and specificity, are crucial performance parameters for application of NGS in clinical practice.

This study attempted to overcome these problems. The aim of this study was (1) to apply genetic analysis using NGS for susceptible MTB strains as well as for drug-resistant strains, and (2) to estimate the PPV and NPV of NGS for detection of drug resistance in clinical practice.

## Methods

### Clinical specimens

This study was approved by the Institutional Review Board of Dongtan Sacred Heart hospital (approval number: HDT NON2017-002). A total of 36 isolated tuberculosis strains were collected at Hallym University Medical Center in 2017. All strains were isolated from clinical specimens. Drug susceptibility patterns were identified in the Korean Institute of Tuberculosis using the absolute concentration method with Lowenstein–Jensen (LJ) medium. This method was performed using the M-kit (Multiplexing MTB drug susceptibility testing [DST] kit; Korean Institute of Tuberculosis, Osong, Korea), which can conduct DST on 16 drugs simultaneously [8, 9]. The critical concentrations for each drug were as follows: isoniazid (INH) 0.2 μg/mL and 1.0 μg/mL, rifampin (RFP) 40 μg/mL, ethambutol (EMB) 2.0 μg/mL, streptomycin (SM) 10 μg/mL, amikacin (AMK) 30 μg/mL, kanamycin (KM) 30 μg/mL, ofloxacin (OFX) 4.0 μg/mL, moxifloxacin (MXF) 2.0 μg/mL, and levofloxacin (LEV) 2.0 μg/mL. The susceptibility for pyrazinamide (PZA) was determined using the pyrazinamidase activity test by Wayne’s method. Strain stock adjusted to McFarland No. 1 was tenfold diluted with phosphate buffered saline. Each 25 μL was inoculated for test wells using repeating pipette. The inoculated media was incubated at 37 °C. During the 1st week, culture conditions were evaluated once daily to identify any contamination. The final interpretation was made in the 4th week to determine the culture status of the controls. Study strains were selected to represent various kinds of drug susceptibility patterns, from all-susceptible to extensively drug-resistant isolates.

### Extraction of nucleic acids

Culture media containing the study strains were boiled at 100 °C for 30 min to inactivate the bacteria. After boiling, the culture media from the liquid culture bottle or colony scrape from the surface of Ogawa media were used for subsequent steps. Nucleic acids were extracted using QIAsymphony DSP Virus/Pathogen Mini kit (Qiagen, Hilden, Germany) according to the manufacturer’s instructions.

### PCR and sequencing

Target genes included in the study were as follows: *katG* and *inhA* for INH resistance, *rpoB* for RFP resistance, *embB* for EMB resistance, *pncA* for PZA resistance, *rpsL* for SM resistance, *eis* for KM/AMK resistance, and *gyrA* for fluoroquinolone (FQ) resistance. All coding regions of the eight target genes responsible for drug resistance were amplified and sequenced by the Ion AmpliSeq TB Research Panel using an IonS5 XL system (Thermo Fisher Scientific, Waltham, MA, USA) according to the manufacturer’s instructions for use [4, 7]. Information about the primers used can be obtained on the webpage provided by Thermo Fisher Scientific (https://ampliseq.com/) or is available from the authors on request.

In brief, the libraries were made using Ion AmpliSeq Library kits (Thermo Fisher Scientific) and labeled by Ion Xpress Barcode Adapter kits (Thermo Fisher Scientific). After purification, the library was amplified using emulsion PCR in an Ion Chef instrument (Thermo Fisher Scientific) and sequenced by an ion-semiconductor sequencer.

### Bioinformatic analysis

Raw sequences from the instrument were aligned to the reference genome of MTB (*Mycobacterium tuberculosis* H37Rv, NC_000962.3) and variants were called using the Ion Report v.5.2 software (Thermo Fisher Scientific) and CLC Genomics Workbench 11 (Qiagen). Coverage was assessed using the coverage analysis plug-ins in the applications. Variants were selected for the final analysis if they were detected in both pipelines and suspected to cause non-synonymous changes (including nonsense, missense, and frame-shift mutations) in coding regions.

### Estimated predictive values using pooled sensitivity and specificity

Data from two previous studies using the same panel that we used were merged with our data to calculate the pooled sensitivity and specificity [4, 7]. PPV and NPV were calculated using the prevalence of drug-resistant MTB [10].

### Statistical analysis

Cohen’s kappa values were calculated to estimate the agreement between the phenotypic and genotypic results. Results with kappa values greater than 0.8 were considered to be almost perfect agreement. Variants were considered to be associated with a resistance phenotype if the *P* value was less than 0.05 in Fisher’s exact test (resistance) or if the variants were found only in resistant strains but not in susceptible strains (possible resistance). All statistical analyses were performed using MedCalc 18.6 (MedCalc Software, Ostend, Belgium).

## Results

### Phenotypic resistance patterns of specimens

Drug resistance patterns determined by absolute concentration methods are displayed in Table 1. Half (n = 18) of the samples showed susceptibility to all drugs tested, while others showed resistance to one or more of the drugs.

**Table 1:** Drug resistance patterns and number of variants in the study samples

Sample number	INH	RFP	SM	EMB	KM	AMK	OFLX	MXF	LFX	PZA	Number of variants
1	S	S	S	S	S	S	S	S	S	S	4
2	S	S	R	S	S	S	S	S	S	S	5
3	R	S	S	S	S	S	S	S	S	S	6
5	S	S	S	S	S	S	S	S	S	S	5
6	R	S	S	S	S	S	S	S	S	S	5
7	S	S	S	S	S	S	S	S	S	S	5
8	S	S	S	S	S	S	S	S	S	S	4
9	S	S	S	S	S	S	S	S	S	S	5
10	S	S	S	S	S	S	S	S	S	S	4
12	S	S	S	S	S	S	S	S	S	S	1
13	S	S	S	S	S	S	S	S	S	S	4
14	S	S	S	S	S	S	S	S	S	S	4
15	S	S	S	S	S	S	S	S	S	S	4
16	S	S	S	S	S	S	S	S	S	S	3
18	S	S	S	S	S	S	S	S	S	S	4
19	S	S	S	S	S	S	S	S	S	S	5
20	S	S	S	S	S	S	S	S	S	S	4
21	R	R	S	S	S	S	S	S	S	S	6
22	S	S	S	S	S	S	S	S	S	S	4
23	R	S	S	S	S	S	S	S	S	S	6
24	R	S	R	S	S	S	S	S	S	S	5
25	R	R	S	S	S	S	R	R	R	R	6
26	S	S	S	S	S	S	R	R	R	S	5
27	R	R	R	R	R	R	S	S	S	R	9
28	S	R	S	R	S	S	S	S	S	S	7
29	R	S	S	S	S	S	S	S	S	S	6
30	R	R	S	R	S	S	S	S	S	R	7
31	R	S	R	S	S	S	S	S	S	S	6
32	R	R	S	R	S	S	S	R	S	R	6
33	S	S	R	S	S	S	S	S	S	S	5
34	S	S	R	S	S	S	S	S	S	S	5
35	R	R	R	R	R	R	S	S	S	R	7
36	R	R	R	R	S	S	R	R	R	R	9
37	S	R	S	S	S	S	R	S	R	R	10
38	R	R	R	R	S	S	R	R	R	R	8
39	R	R	R	S	S	S	S	S	S	S	6

*R* resistance, *S* susceptible, *INH* isoniazid, *RFP* rifampin, *SM* streptomycin, *EMB* ethambutol, *KM* kanamycin, *AMK* amikacin, *OFLX* ofloxacin, *MXL* moxifloxacin, *LFX* levofloxacin, *PZA* pyrazinamide

### Sequencing results

The sequencing experiments were successfully performed for all samples included in the study. Coverage at 1×, 20×, 100×, and 500× were 99.57% to 100%, 96.92% to 100%, 85.71% to 100%, and 45.24% to 96.62%, respectively. Because all samples showed more than 96.92% for 20× coverage and 100% in 27 samples, our experiments could be considered to cover virtually all target regions. Meanwhile, some samples showed low coverage for specific regions in parts of target genes, which will be discussed later.

### Variant interpretation and genotype–phenotype correlation

A total of 39 variants were found in the samples. The number of variants in each sample ranged from one to ten (Table 1). Table 2 shows the list of variants found in our study and their interpretations. In total, 24 variants were determined to be related to resistance to the corresponding drugs (3 for *gyrA*, 2 for *rpsL*, 5 for *embB*, 3 for *inhA*, 3 for *katG*, 4 for *pncA*, and 3 for *rpoB*), and the remaining 15 variants were thought to be benign polymorphisms. Among the variants associated with resistance phenotypes, six have not yet been reported (*katG* L378R and Y597D; *pncA* S18Ter and H82Pfs; *embB* I419V; and *rpoB* R552L).

**Table 2:** List of variants found in study strains and their clinical significance

Gene	Related drug	Nucleotide change	Suspected amino acid changes	No. of strains having variant among sensitive strains (%)	No. of strains having variant among resistant strains (%)	P value*	Previous report	Interpretation
*gyrA*	FQ	G61C	E21Q	30/30 (100)	6/6 (100)	> 0.05	Yes [22]	Susceptible
*gyrA*	FQ	C269T	A90V	0/30 (0)	2/6 (33.3)	0.024	Yes [23]	Resistance
*gyrA*	FQ	G280A	D94N	0/30 (0)	1/6 (16.7)	0.17	Yes [23]	Possible resistance
*gyrA*	FQ	A281G	D94G	0/30 (0)	1/6 (16.7)	0.17	Yes [23]	Possible resistance
*gyrA*	FQ	G284C	S95T	29/30 (96.7)	6/6 (100)	> 0.05	Yes [16]	Susceptible
*gyrA*	FQ	C846G	N282K	3/30 (10)	1/6 (16.7)	0.17	No	Susceptible
*gyrA*	FQ	G2003A	G668D	29/30 (96. 7)	6/6 (100)	> 0.05	Yes [22]	Susceptible
*rpsL*	SM	A128G	K43R	0/26 (0)	3/10 (30)	0.017	Yes [24]	Resistance
*rpsL*	SM	A262C	K88Q	0/26 (0)	1/10 (10)	0.298	Yes [24]	Possible resistance
*embB*	EMB	A916G	M306V	1/29 (3.4)	3/7 (42.9)	0.018	Yes [16]	Resistance
*embB*	EMB	G918A	M306I	0/29 (0)	2/7 (28.6)	0.033	Yes [16]	Resistance
*embB*	EMB	A916C	M306L	0/29 (0)	1/7 (14.3)	0.194	Yes [16]	Possible resistance
*embB*	EMB	A956C	Y319S	0/29 (0)	1/7 (14.3)	0.22	Yes [16]	Possible resistance
*embB*	EMB	A1061D	D354A	1/29 (3.4)	0/7 (0)	> 0.05	Yes [25]	Susceptible
*embB*	EMB	A1255G	I419V	0/29 (0)	1/7 (14.3)	0.194	No	Possible resistance
*inhA*	INH	T62C	I21T	0/21 (0)	1/15 (6.7)	> 0.05	Yes [16]	Possible resistance
*inhA*	INH	T74C	I25T	0/21 (0)	1/15 (6.7)	> 0.05	Yes [26]	Possible resistance
*inhA*	INH	T280G	S94A	0/21 (0)	2/15 (13.3)	0.167	Yes [16]	Possible resistance
*katG*	INH	923_924delinsAG	T308L	1/21 (4.8)	0/15 (0)	> 0.05	No	Susceptible
*katG*	INH	C944G	S315T	0/21 (0)	5/15 (33.3)	< 0.05	Yes [16]	Resistance
*katG*	INH	C944T	S315N	0/21 (0)	1/15 (6.7)	> 0.05	Yes [16]	Possible resistance
*katG*	INH	A1133C	L378R	0/21 (0)	1/15 (6.7)	> 0.05	No	Possible resistance
*katG*	INH	C1317A	Q439H	1/21 (4.8)	0/15 (0)	> 0.05	No	Susceptible
*katG*	INH	C1388A	R463L	16/21 (76.2)	12/15 (80)	> 0.05	Yes [16]	Susceptible
*katG*	INH	G1595A	A532V	1/21 (4.8)	0/15 (0)	> 0.05	No	Susceptible
*katG*	INH	G1715A	T572M	1/21 (4.8)	0/15 (0)	> 0.05	No	Susceptible
*katG*	INH	A1789C	Y597D	0/21 (0)	1/15 (6.7))	> 0.05	No	Possible resistance
*katG*	INH	T1873C	T625A	1/21 (4.8)	2/15 (13.3)	> 0.05	No	Susceptible
*pncA*	PZA	C53A	S18Ter	0/28 (0)	1/8 (12.5)	> 0.05	No	Possible resistance
*pncA*	PZA	A139C	T47P	0/28 (0)	1/8 (12.5)	> 0.05	Yes [16]	Possible resistance
*pncA*	PZA	241insT	H82Pfs	0/28 (0)	1/8 (12.5)	> 0.05	No	Possible resistance
*pncA*	PZA	T254G	L85R	0/28 (0)	1/8 (12.5)	> 0.05	Yes [16]	Possible resistance
*rpoB*	RFP	C38T	P13L	1/25 (4)	0/11 (0)	> 0.05	No	Susceptible
*rpoB*	RFP	C1333T	H445Y	0/25 (0)	1/11 (9.1)	> 0.05	Yes [27]	Possible resistance
*rpoB*	RFP	C1349T	S450L	0/25 (0)	10/11 (90.9)	< 0.001	Yes [27]	Resistance
*rpoB*	RFP	T1355C	L452P	1/25 (4)	0/11 (0)	> 0.05	Yes [27]	Susceptible
*rpoB*	RFP	G1655T	R552L	0/25 (0)	1/11 (9.1)	> 0.05	No	Possible resistance
*rpoB*	RFP	G1882T	A628S	1/25 (4)	0/11 (0)	> 0.05	No	Susceptible
*rpoB*	RFP	A3305C	K1102T	1/25 (4)	0/11 (0)	> 0.05	No	Susceptible

*FQ* fluoroquinolones, *SM* streptomycin, *EMB* ethambutol, *INH* isoniazid, *PZA* pyrazinamide, *RFP* rifampin

* P value resulting from Fisher’s exact test

The overall agreements between genotypic and phenotypic results are shown in Table 3. For INH, RFP, and EMB, the two tests showed almost perfect agreement (Cohen’s kappa of 0.824 to 1.000), while for SM, the agreement was poor (Cohen’s kappa of 0.491).

**Table 3:** Agreement between phenotypic and genotypic test results

Genotypic result	Phenotypic result (N)		Cohen’s Kappa (95% confidence interval)
	Resistant	Susceptible	
INH			
Mutated (N = 12)	12	0	0.824 (0.632–1.000)
Unmutated (N = 24)	3	21	
RFP			
Mutated (N = 11)	11	0	1.000 (1.000–1.000)
Unmutated (N = 25)	0	25	
EMB			
Mutated (N = 8)	7	1	0.916 (0.753–1.000)
Unmutated (N = 28)	0	28	
PZA			
Mutated (N = 4)	4	0	0.609 (0.247–0.970)
Unmutated (N = 4)	4	28	
SM			
Mutated (N = 4)	4	0	0.491 (0.118–0.683)
Unmutated (N = 32)	6	26	
AMK, KM			
Mutated (N = 0)	0	0	N/A
Unmutated (N = 36)	2	34	
FQ			
Mutated (N = 4)	4	0	0.769 (0.458–1.000)
Unmutated (N = 32)	2	30	

*INH* isoniazid, *RFP* rifampin, *EMB* ethambutol, *PZA* pyrazinamide, *SM* streptomycin, *AMK* amikacin, *KM* kanamycin, *FQ* fluoroquinolones

### Estimated predictive values using pooled sensitivity and specificity

The calculated pooled sensitivity and specificity are displayed in Table 4. Generally, NPVs were remarkably high (greater than 97%), while the PPVs were variable, ranging from 44% to 100%. The PPV for AMK/KM could not be estimated because the pooled sensitivity was 0%.

**Table 4:** Pooled analytical performances of the Ion AmpliSeq TB Research Panel for detecting of drug resistances

Drug	Pooled sensitivity (%) (95% CI)	Pooled specificity (%) (95% CI)	Prevalence of drug resistance [10] (%)	PPV (%) (95% CI)	NPV (%) (95% CI)
INH	91.3 (82.0–96.7)	100.0 (86.3–100.0)	15.5	100.0 (100.0–100.0)	98.4 (96.7–99.3)
RFP	98.0 (89.1–99.9)	98.0 (89.6–100.0)	9.3	83.7 (42.4–97.3)	99.8 (98.5–100.0)
EMB	96.7 (87.8–99.9)	90.0 (80.5–95.9)	6.7	41.0 (25.5–58.4)	99.7 (98.2–100.0)
PZA	65.0 (40.8–84.6)	96.3 (89.4–99.2)	4.3	43.8 (19.7–71.2)	98.4 (97.1–99.1)
SM	62.1 (42.3–79.3)	98.6 (92.4–100.0)	5.4	71.6 (26.0–94.7)	97.9 (96.6–98.6)
AMK, KM	0.0 (0.0–70.8)	100.0 (96.3–100.0)	2.2	N/A	97.8 (97.8–97.8)
FQ	66.7 (34.9–90.1)	100.0 (95.9–100.0)	2.9	100.0 (100.0–100.0)	99.0 (97.8–99.6)

*CI* confidence interval, *PPV* positive predictive value, *NPV* negative predictive value, *INH* isoniazid, *RFP* rifampin, *EMB* ethambutol, *PZA* pyrazinamide, *SM* streptomycin, *AMK* amikacin, *KM* kanamycin, *FQ* fluoroquinolones, *N/A* not applicable

## Discussion

One of the biggest issues with drug susceptibility tests for MTB is their turnaround time. Traditional methods such as absolute concentration or proportional methods usually take up to several weeks. This issue is inherent in phenotypic methods, and arises from the low growth rate of MTB. Genotypic methods have overcome this issue, with turnaround times of several days [2].

There have been many reports of using NGS for MTB drug susceptibility tests, and some of them have revealed the utility of semiconductor-based NGS [4, 5, 7]. Two of these reports used the same panel as in our study. However, they applied this technology to MTB isolates resistant to one or more drugs, which is unlikely to be encountered in routine clinical practice. For clinical application of NGS to drug susceptibility testing, the test should provide results in advance of phenotypic testing, without any information regarding the susceptibility. Therefore, we need to have knowledge of results from susceptible strains as well as from resistant strains. In this study, we used 18 susceptible strains to reveal the benign polymorphisms.

In this study, Cohen’s kappa values were calculated to assess the agreement between phenotypic and genotypic test results. Resistance patterns for INH, RFP, and EMG showed almost perfect agreement between the two methods, although the agreements were poor for other drugs. In comparison to the results from previous studies, genotypic tests showed relatively poor performance for SM (0.491 versus 0.769, 0.746) [4, 7]. However, the 95% confidence interval was wide due to the small number of specimens, and we therefore cannot conclude that our results were statistically different from those of other studies.

Fisher’s exact test was used for determine the clinical significance of each variant. Among 39 variants found in this study, six (*gyrA* c.C269T, *rpsL* c.A128G, *embB* c.A916G and c.G918A, *katG* c.C944G and *rpoB* c.C1349T) showed P values less than 0.05 and were designated as ‘resistance’. ‘Possible resistance’ variants were determined as those found only in resistance strains and having P values greater than 0.05. Based on our findings, 18 variants (*gyrA* c.G180A and c.A281G, *rpsL* c.A262C, *embB* c.A916C, c.A956C, and c.A1255G, *inhA* c.T62C, c.T74C, and c.T280G, *katG* c.C944T, c.A1133C, and c.A1789C, *pncA* c.C53A, c.A139C, c.241insT, and c.T254G, and *rpoB* c.C1333T and c.G1655T) were allocated in this category.

Most instances of RFP resistance come from mutations in the “rifampicin resistance-determining region” of *rpoB*, spanning codons 507–533 [11, 12]. In contrast, the most frequent variant found in *rpoB* in our study was S450L (10 strains), and no variants were found in codons 507–533. This discrepancy was probably due to the relatively low coverage at these regions (< 100×) in many samples.

Two main causative genes for INH resistance are *katG* and *inhA*. The most prevalent variant in this study was *katG* R463L, which was determined to be a benign polymorphism. Among variants associated with the resistance phenotype, the *katG* S315T mutation was most frequently found, which is consistent with previous reports [11, 13]. However, the most prevalent mutation in the promoter region of *inhA* c.− 15C>T could not found because the Ion AmpliSeq panel did not cover the area [4, 7, 11]. This missing coverage and other genes responsible for INH resistance, such as *ahpC*, might be the reason that no mutations were found in three strains with resistance phenotypes.

Codon 306 in *embB* is the most important area for EMB resistance [14, 15]. This study showed similar results: six out of seven resistant strains showed variants in codon 306. Interestingly, one strain with the M306V variant was determined to be susceptible to EMB in phenotypic assays. This discrepancy might be due to a failure in the phenotypic assay or the presence of other mechanisms interacting with the *embB* mutations. We could not further investigate this issue, which should be the subject of subsequent studies.

We have found two novel loss-of-function mutations in *pncA* that are responsible for PZA resistance. There have already been many frame-shift mutations reported in the *pncA* gene [16]. Pyrazinamidase, the product of the *pncA* gene, is required to convert PZA to its active form, pyrazinoic acid [17]. Therefore, it is reasonable to consider that frame-shift mutations or nonsense mutations in the *pncA* gene cause drug resistance. No causative mutations were detected in four out of eight resistant strains. This result is comparable to those of previous studies, suggesting the presence of mutations in promoter and/or other regulatory genes [17, 18].

Only four mutations could be found in *rpsL*, and none in *eis*. For *rpsL*, the most common mutation is K43R [11], which was also found in this study. Meanwhile, the agreements between phenotypic and genotypic results were regrettably poor for these drugs, which researchers and/or doctors should be alert to when using this panel. There are many other genes responsible for resistance to these drugs, such as *rrs*, which might explain the poor performances [19].

FQ resistance arises from mutations in *gyrA* and/or *gyrB*, with codons 90 and 94 in *gyrA* most frequently involved [20, 21]. Our data showed similar results, and all variants other than those in codons 90 and 94 were determined to be benign polymorphisms. Specifically, three variants, E21Q, S95T, and G668D, were found in almost every strain, suggesting the existence of common polymorphisms in MTB in Korea.

We estimated pooled sensitivity and specificity using combined data from this study and previous studies [4, 7]. The NPVs ranged from 97.8 to 99.8%, while the PPVs were variable, from 43.8 to 100%. These data suggested that NGS can be used as a tool to rule out drug resistance before phenotypic results are available.

## Conclusions

In conclusion, we successfully applied NGS to the genetic analysis of drug resistance in MTB, as well as in susceptible strains. The Ion AmpliSeq Tb Panel showed satisfactory specificity and NPV and can therefore be used for early exclusion of resistance in MTB. The panel need to be enhanced to increase the sensitivity and PPV. A useful result of this study was a list of variants with their clinical significance: six variants with confirmed resistance, 18 with possible resistance, and 15 that were susceptible. This list could be used as a guide in future applications of NGS in mycobacteriology laboratories. When analyzing NGS results, the coverage analysis of each sample for each gene, as well as benign polymorphisms not related to drug resistance, should be considered.

## Abbreviations

Tb: tuberculosis
MTB: *Mycobacterium tuberculosis*
NGS: next-generation sequencing
INH: isoniazid
RFP: rifampin
EMB: ethambutol
SM: streptomycin
AMK: amikacin
KM: kanamycin
OFX: ofloxacin
MXF: moxifloxacin
LEV: levofloxacin
PZA: pyrazinamide
FQ: fluoroquinolone
PPV: positive predictive value
NPV: negative predictive value
